# Translation, cross-cultural adaptation and validation of the EPOCH-RRT questionnaire “Empowering Patients On Choices For Renal Replacement Therapy” for the Brazilian context

**DOI:** 10.1590/2175-8239-JBN-2021-0224en

**Published:** 2022-06-27

**Authors:** Luciana Adorno Sattin Rodrigues, Fernando Antonio de Almeida, Cibele Isaac Saad Rodrigues

**Affiliations:** 1 Pontifícia Universidade Católica de São Paulo, Faculdade de Ciências Médicas e da Saúde, Programa de Estudos Pós-graduados em Educação nas Profissões de Saúde, Sorocaba, SP, Brasil.

**Keywords:** Surveys and Questionnaires, Translations, Validation Study, Nephrology, Renal Replacement Therapy, Hemodialysis Units, Hospital, Inquéritos e Questionários, Traduções, Estudo de Validação, Negrologia, Terapia de Substituição Renal, Unidades Hospitalares de Hemodiálise

## Abstract

**Introduction:**

Chronic kidney disease (CKD) is a global public health problem. In Brazil, the incidence and prevalence rates of dialysis CKD progressively increase, but the transition process is a challenge for patients and caregivers in coping with the disease. Dialysis urgency, lack of planned access or prior knowledge of treatment is a reality for most. Guidelines recommend that treatment options should include the conscious preference of a fully informed patient. However, pre-dialysis educational information is an exception, leading to a large number of unplanned initial dialysis. The original study “Empowering Patients on Choices for Renal Replacement Therapy” (EPOCH-RRT) aimed to identify patient priorities and gaps in shared decision-making about dialysis, using structured interviews with questions about demographics, clinical history and patients' perception of their health. The goal of this study was to carry out the translation, cross-cultural adaptation and validation of the questionnaires used in the EPOCH-RRT Study for the Brazilian context.

**Method:**

This is a methodological study that consisted of the initial translation, synthesis of the translations, back translation, review by a committee of experts, pre-test and evaluation of the psychometric properties of the instrument. All ethical precepts were followed.

**Results:**

The questionnaires were translated, adapted and validated for the Brazilian context. Additionally, it was applied to 84 chronic renal patients on hemodialysis, peritoneal dialysis and outpatients.

**Discussion:**

There is a lack of an educational-therapeutic approach aimed at patients with CKD, and the EPOCH-RRT questionnaire can be a tool for Brazilian dialysis services to change this paradigm.

## Resumo

**Introdução:**

A doença renal crônica (DRC) é um problema de saúde pública mundial. No Brasil, as taxas de incidência e prevalência da DRC dialítica aumentam progressivamente, mas o processo de transição apresenta-se como desafio para pacientes e cuidadores no enfrentamento da doença. Urgência dialítica, ausência de acesso planejado ou conhecimento prévio do tratamento é uma realidade para a maioria. Diretrizes recomendam que opções de tratamento devam incluir a preferência consciente de um paciente totalmente informado. No entanto, informação educacional pré-diálise é exceção, acarretando grande número de diálises iniciais não planejadas. O estudo original “Empowering Patients on Choices for Renal Replacement Therapy” (EPOCH-RRT) teve por objetivo identificar as prioridades do paciente e as lacunas na tomada de decisões compartilhadas sobre a diálise, utilizando entrevistas estruturadas, com questões sobre dados demográficos, história clínica e percepção dos pacientes sobre sua saúde. O objetivo desta pesquisa foi realizar a tradução, adaptação transcultural e validação dos questionários utilizados no Estudo EPOCH-RRT para o contexto brasileiro.

**Método:**

Trata-se de estudo metodológico que consistiu na tradução inicial, síntese das traduções, retro tradução, revisão por um comitê de especialistas, pré-teste e avaliação das propriedades psicométricas do instrumento. Todos os preceitos éticos foram seguidos.

**Resultados:**

Os questionários foram traduzidos, adaptados e validados para o contexto brasileiro. Adicionalmente, foi aplicado em 84 pacientes renais crônicos em hemodiálise, diálise peritoneal e ambulatoriais.

**Discussão:**

Há carência de enfoque educativo-terapêutico dirigido aos pacientes com DRC, e o questionário EPOCH-RRT pode ser uma ferramenta para serviços de diálise brasileiros mudarem esse paradigma.

## Introduction

Chronic kidney disease (CKD) is a worrying public health problem worldwide that affects 10% to 15% of the adult population^1^. In the United States, between 1988 and 2002, the prevalence of reduced filtration rate (< 60 mL/min/ 1.73 m^2^ or stages 3 to 5) increased from 4.7% to 6.5%. From 2003 to 2012, the prevalence stabilized at 6.4 to 6.9^2^.

In Brazil, according to the Brazilian Dialysis Census of the Brazilian Society of Nephrology, in the period of 2009-2018 there was a progressive increase in the number of prevalent patients in a chronic dialysis program, with an average annual increase of 5,587^3^. The 2020 census shows consistency in the increase in the number of dialyzed patients to 144,779, representing 44,264,000 new patients that year^4^.

At stage 5, the patient is indicated to a program or to start renal replacement therapy (RRT), and this process of transition from CKD to end-stage renal disease presents itself as a significant challenge for patients and caregivers in coping with the disease and its unpredictable consequences^5^.

Although RRT enables us to prolong life, it triggers unexpected and conflicting situations, compromising daily life and quality of life of the patient, their families and caregivers, imposing adaptations and changes to their lifestyles, so it is necessary to share this confrontation with the family or with other people to seek help.

Changes in patients’ lives are uncomfortable and continuous. They may feel different, excluded or stigmatized by dietary and water restrictions; having vascular access; needing multiple medications; and undergoing dialysis, determining a high prevalence of psychological disorders and a perception of a definitive and negative impact on their lives.

However, family structures are not always able to support these situations. They need the support of healthcare professionals, able to meet the emotional demands of these individuals, as well as support and collaborate with other people in the community^6^. A study with specialist nurses in Nephrology from Sweden showed that emotional support to the patient during the transition to dialysis may be lacking, so education, local and national, is necessary for nurses to be able to provide this emotional support^7^.

We understand the importance of socio-educational activities for these patients so that they have greater knowledge about their CKD, acquire security and greater subsidies for self-care and, in addition to better adhere to treatment, indispensable for improving the quantity and quality of life^8^.

In Brazil, most individuals initiate RRT under urgent or emergency dialysis basis, without planned access and without information. The study by Peixoto et al., in Belo Horizonte, showed that 70% of patients started dialysis in an unplanned way^9^.

A systematic review of qualitative studies pointed out that studies and Guidelines from different continents recommend that the choice between treatment options for CKD should respect the conscious preference of a fully informed patient^10^.

We believe that encouraging patients to take a more active role in their own healthcare may increase the time to start dialysis; provide fewer hospitalizations; improve the quality of life and the efficiency of treatment, as well as its results; with lower costs to the healthcare system^11,12^.

However, only a minority of CKD patients receive pre-dialysis education and initiate RRT on a planned basis. An international integrative review study showed that between 24% and 49% of patients start dialysis unplanned, without access to minimal knowledge^13^.

Knowledge about the factors associated with the decision to choose the treatment modality for CKD can provide evidence to the multidisciplinary team involved, to improve the way of educating patients and their families, favoring adequate communication, patient and family/caregiver involvement in shared decision making^14^.

In the Empowering Patients on Choices for Renal Replacement Therapy (EPOCH-RRT) study^15^, with the aim of identifying patient priorities and gaps in shared decision-making, three different interview protocols for non-dialysis CKD (CKD-ND) were developed for HD and PD patients. The protocols used a mixed methods approach, with open and closed questions. The protocols were tested in interviews with 179 patients with stage 5 CKD in the beginning^16^.

Afterwards, a retrospective quantitative study was carried out to evaluate the decision-making process of the dialysis modality and the impact on the daily lives of 1963 people undergoing HD or PD. Based on the interviews and the study, the DA (“Decision Aid”) was developed and then its effectiveness was measured with pre- and post-evaluation.

The multidimensional instrument developed subsequently proved to be effective in increasing knowledge and reducing decision-making conflicts^17^.

The lack of an educational-therapeutic approach, built with the participation of interested parties, is also observed in Brazilian dialysis services, added to the fact that there are still few studies on the subject, led us to carry out this research^18^, to translate the protocols used in the EPOCH-RRT Study and verify if the results are reproduced in a Brazilian study.

The objectives of the present study were to translate, cross-culturally adapt and validate the interview protocols “Interview Protocol for Hemodialysis Patients”, “Interview Protocol for Peritoneal Dialysis Patients” and “Interview Protocol for Chronic Kidney Disease Patients Not on Dialysis” used in the study “Empowering Patients on Choices for Renal Replacement Therapy (EPOCH - RRT)” for the Brazilian context.

## Methods

This is a methodological study of translation, cross-cultural adaptation and validation for the Portuguese-speaking population of Brazil^16^.

**Figure 1:**
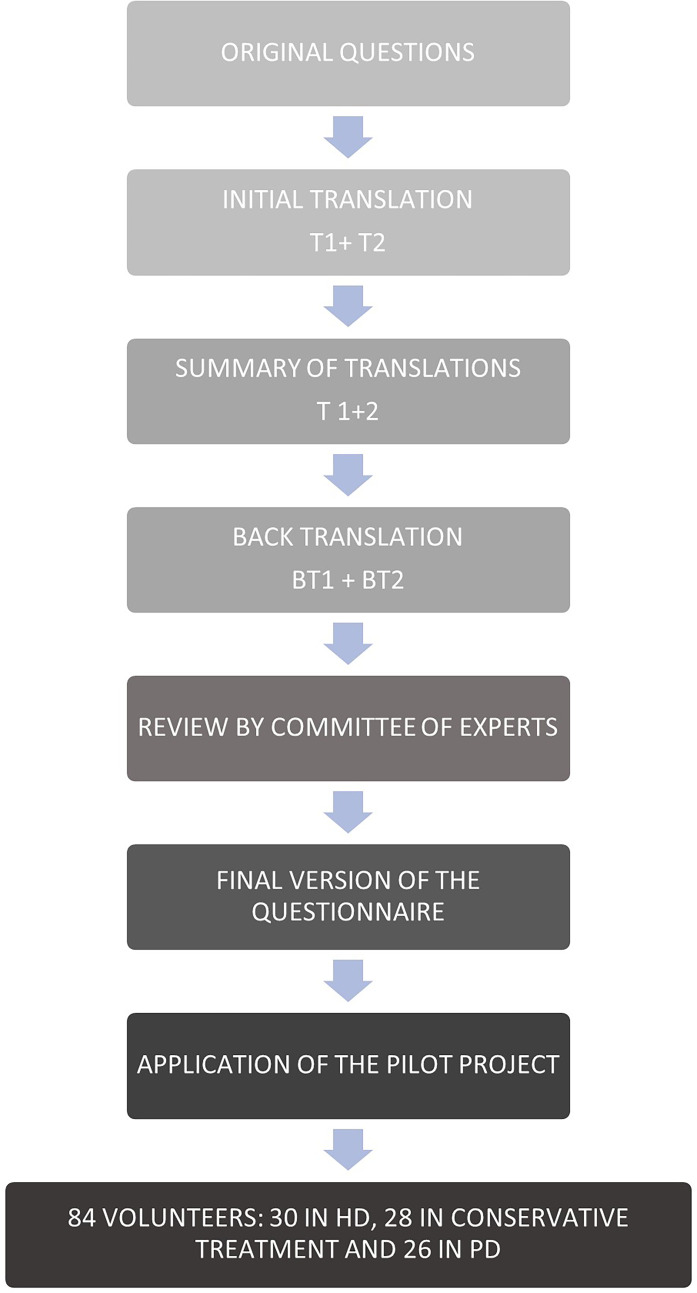
Methodology.

Figure 1 depicts the synthesis of the methodological steps followed.

So far, there is no similar protocol in our language and the current trend proposes the use of validated instruments as opposed to the development of new scales, allowing further comparisons between different countries, in a safe and reliable way.

The protocols included questions for sociodemographic characterization, clinical history and patients’ perception of their health. Permission was obtained from PCORI - Patient-Centered Outcomes Research Institute and lead author Francesca Tentori, MD., PhD.

The process of cross-cultural adaptation of an instrument consists of 2 basic components: literal translation of words and sentences from one language to another and adaptation to the cultural context of the population in the target culture^19^. It usually comprises six types of equivalences: conceptual, of items, semantics, operational, measurement and functional^20^.

### Initial Translation

Two translations of the instrument were made from the original language (English) to the target language (Brazilian Portuguese). The two translations were performed independently by a nephrologist professor fluent in the language and with a postdoctoral degree from New York (USA) and an experienced English professor, of Brazilian nationality. The translators independently produced a translated version of the questionnaires^21^.

Translator 1 (T1). This translator was aware of the concepts to be examined in the questionnaire. Its adaptations are intended to provide equivalence in a more clinical and more reliable way from a measurement perspective.

Translator 2 (T2). This translator not informed of the concepts to be translated and was unfamiliar with the topic. This is the naive translator, more likely to detect different meanings from the original. It is less influenced by an academic objective and offers a translation that reflects the language used by the lay population^21^.

### Synthesis of Translations

The two translators and a recording observer (the researcher, also fluent in English) met to synthesize the results of the translations. They made a synthesis of the two translations, producing a common translation, called T1+2, with a written report documenting the entire process.

### Back Translation

Process used for the cross-cultural adaptation of research instruments, particularly questionnaires. A first version is translated back into its source language and this back-translation is compared with the original. Differences between back-translation and the source text may indicate problems to be overcome in relation to the first translation, considered equivalence failures^22^.

Thus, from the synthesis of the questionnaire translation, and blinded to the original version, two translators (B1 and B2), who did not participate in the first stage, made the version of the questionnaire back into English. This is a validity checking process to ensure that the translated version reflects the content of the original version consistently and reliably. The back-translations were produced by two sworn translators accredited by JUCESP (B1 and B2), avoiding information bias and increasing the probability of finding imperfections^21^.

### Expert Committee Review

The composition of a committee aims to achieve cross-cultural equivalence. Its role is to consolidate all versions of the questionnaire and develop the preliminary version of the questionnaire for field testing. The committee should review all translations and reach consensus on discrepancies. The material made available to committee members included the original questionnaire and all translations (T1, T2, T1+2, RT1, RT2), with the corresponding reports. It is incumbent upon the committee to provide written documentation of the issues and the reasons used to reach the final decision on them. Decisions were made to achieve equivalence between the source and target version in four areas: semantic, idiomatic, experiential and conceptual, whose concepts are well established in the literature^19^.

In this study, the committee was composed of the two translators (T1 and T2), the researcher and the advisor, a professor of Bioethics at the graduate level and a nephrologist. After acceptance, the original questionnaires, the consensual version translated into Portuguese and the back-translations were sent individually via e-mail. This committee, in a meeting, produced the final version of the questionnaires.

### Validation

To have international quality, the instrument, after translation and back-translation, and application of the pilot project, underwent a psychometric study, in which its reliability and validity were investigated^19,23^.

Adequate instruments have properties established by the principles of psychometry, a science that measures responses to phenomena that are not easily quantifiable. Good tools are those that are reliable and have appropriate acceptance by the interviewees; developed and validated for the purpose proposed in the investigation, they are capable of distinguishing patients with and without the problem and of measuring minimal significant changes. We use the following concepts^24^.

### Reliability, Reproducibility or Precision

Reliability is the degree of consistency, coherence or precision with which the instrument measures an attribute^25^. It can be evaluated through internal consistency and reproducibility.

Internal consistency represents the average of the correlations between all its items. It can be measured by Cronbach’s alpha coefficient, measuring the homogeneity of items within the same domain^26^.

Reproducibility assesses the degree to which an instrument achieves stable results in a short time interval between measurements, assuming that there has been no clinical change (test - retest). It can be obtained by evaluating the agreement between two or more observers (inter-observer reliability) and through the agreement between observations made by an observer (intra-observer reliability) on different occasions or by an instrument in different scenarios. For analysis, the evaluation by Kappa and intraclass correlation coefficients was used^20,27-30^.

### Validity or Accuracy

Refers to the degree of accuracy of the result of a measurement. There are three types: content, criterion and construct^20,31^.

### Content Validity

Validity is the degree to which the contents included in the questionnaire or scale are adequate to what one wishes to measure^32^.

We used the Content Validity Index (CVI) or agreement between judges, which assesses the representativeness of the measure in relation to the content addressed, by dividing the number of judges who judged the item with a score of extreme relevance or relevant by the total number of judges (CVI for each item), which resulted in the proportion of judges who judged the item to be valid. The Committee of Judges was composed of 10 bilingual healthcare professionals who agreed to voluntarily fill out the questionnaire^33^.

### Criterion Validity

There is criterion validity if it is coherently related to other different measures that assesses the same phenomenon.

It confirms the correlation between the scores of the instrument to be tested and some external criterion. There are two types: concurrent validity, which is obtained by applying two similar instruments, at the same time to the same individuals for later comparison, and predictive validity, which refers to the accuracy of the instrument in predicting future events^25^.

In this study, criterion validity was not used because there is no other questionnaire that assesses the same phenomenon.

### Construct Validity

Construct validity is the fundamental form of validity of psychological instruments. It can be measured through internal analysis (exploratory, confirmatory factorial) and external analysis, based on clinical and demographic criteria derived from clinical experience and medical practice, and also by correlating with other existing instruments, ideally the gold standard^20^.

As there are no questionnaires or similar scales for correlation, we chose to analyze internal consistency using Cronback’s Alpha coefficient, which is consistent when exceeding 0.70 for each questionnaire^32,34^.

### Questionnaires Application

To apply the questionnaires the questionnaires, we received authorization from: the Institutional and from the author; from the dialysis units involved (A, B and C), and from the Research Ethics Committee of the Faculty of Medical and Healthcare Sciences of the Pontifical Catholic University of São Paulo (CAAE: 19647119.0.0000.5373).

The period of application of the questionnaires was from the second half of 2019 to the first half of 2021, in three of the four TRS units in the municipality of Sorocaba.

A total of 84 volunteers were included in the pilot project, 30 on HD, 26 on PD and 28 on conservative treatment. The inclusion criteria were adults with CKD (> 18 years), on HD or PD for at least 90 days, without restrictions on gender, race or underlying disease, in a possible universe of 657 individuals, who had no functional problem that prevented them from participating.

### Statistical Analysis

In order to validate the three EPOCH-RRT questionnaires, we estimated the Cronbach’s Alpha and Kappa coefficients for each one.

For Cronbach’s Alpha, the calculation was performed only for the specific questions about kidney disease and treatment, as the general questions are not numerically standardized to represent a quantitative profile of the issue studied. We assessed the Cronbach’s Alpha only for the questionnaires for HD and PD patients, as the more qualitative questionnaire for patients without dialysis did not enable this analysis to be reliably performed.

We assessed all the questions in the Kappa coefficient. This is the analysis of the responses to the questionnaires using the selected evaluators described above.

The total sample of individuals was considered satisfactory and sufficient to perform exploratory analysis, statistical tests of hypotheses, comparison and association between the groups.

### Kappa Coefficient

According to the reference by Fonseca et al.^35^, the Kappa coefficient is used to estimate the agreement between examiners to answer a questionnaire. For this calculation, several examiners need to assign scores to the questionnaire as an experiment, and then the difference between the answers of each one is estimated to test if everyone’s interpretation is consistent.

In the present study, 10 judges evaluated all the questions of the three questionnaires according to the patients who had answered the questions. The Kappa coefficient classification reference is that of Landis and Koch^36^. To obtain substantial agreement, the result must be > 0.60 to 0.80, and perfect agreement is considered between 0.81 and 1.0.

### Cronbach’s Alpha

It aims to validate instruments by evaluating whether all items measure the same construct. It is a reliability index best applicable to scales that are not dichotomous, working most accurately for questions with more than three possible numerical answer choices.

Questions with numerical answers can be standardized to obtain a corrected index, because if the scales are very different, this fact directly interferes with the calculation, making it a questionnaire with less reliability because of the large variations in answers. When the scale is: the higher the score, the more positive the answer, the other questions need to follow the same logic. Otherwise, we must change the coding so that the higher the scale value, the more positive the response.

In the non-dialysis questionnaire, general health originally had a scale in which the higher the number, the worse the patient’s health, while the stage of choice showed that the higher the number, the more decided the patient was about treatment. Therefore, we inverted the overall health variable so that the higher the number, the better the health. Cronbach’s Alpha based on standardized items was used in the study to smooth out differences in scale.

Reference values to estimate whether the reliability index is acceptable are: > 0.7 is acceptable for a validated questionnaire, the value between 0.6 and 0.7 has low reliability. If the Alpha is > 0.95, it may indicate that the questions are redundant rather than reliable^37^.

We used Microsoft Excel 2016 to organize the data, and the IBM SPSS (Statistical Package for the Social Sciences) version 22 to calculate percentages, mean values, standard deviation and statistical tests.

## Results

### Calculation of Cronbach’s Alpha and Kappa’s Coefficient

Cronbach’s Alpha measures the reliability of the three questionnaires on the specific questions regarding kidney disease. The Alpha value was also presented in case the researcher removed a question from the questionnaire. We applied the Kappa coefficient to all questions.

Those patients under conservative treatment (n = 28) answered two specific questions about CKD, reporting how they felt about their general health and at what level of decision they were about the choice of treatment. It makes no sense to use Cronbach’s Alpha for these qualitative questions.

There are only two questions with little variability in the choice stage answers (variance < 1) and greater variation in the answers about general health; therefore, reliability is little because it is estimated that the result could be different if applied to another group of patients under conservative treatment.

If only one question remains, the Alpha coefficient cannot be calculated.

The Kappa coefficient showed agreement in question 1 (p-value: 0.149), question 7 (p-value: 0.086), question B3 (p-value: 0.086), question B7 (p-value: 0.018) and question B9 (p-value: 0.140). In the other questions, the agreement was classified as perfect, as it reached a p-value = 1; or there was no calculation possible because all the scores were identical, indicating total agreement among the judges.

### Peritoneal Dialysis

The 26 PD patients answered eight questions regarding CKD and obtained Cronbach’s Alpha equal to 0.791, which can be classified as concordant, and very close to 0.8. The summary of how much Cronbach’s Alpha would be, if we removed a single question (item), follows in Table 1.

**Table 1:** Cronbach’s alpha for epoch-rrt “interview protocol for patients with chronic kidney diseases on peritoneal dialysis”

Statistics per item - Questionnaire for patients under PD (specific kidney questions)				
**Item**	**Mean value if the item is deleted**	**Variance if the item is deleted**	**Correlation corrected if the item is deleted**	**Cronbach’s alpha if the item is deleted**
Q 1_General_Health	35.42	243.85	0.489	0.790
Q 9_Stomach_full	33.23	197.79	0.624	0.749
Q 9_Tired_always	33.46	200.18	0.545	0.760
Q 9_Problems_sleep	32.58	200.73	0.414	0.785
Q 9_Iching_dry_skin	32.12	198.59	0.551	0.759
Q 9_Shortness_of_breath	34.42	191.05	0.572	0.755
Q 9_Lacking_apetite	34.15	184.38	0.677	0.736
Q 9_Pain_stomacho	34.92	212.95	0.319	0.799

That is, if you remove the question about stomach pain, the Cronbach’s Alpha of the questionnaire would be 0.799. The remaining items would form another table like this one, in which it would be possible to check if the index would increase with the removal of some variable or decrease, showing that the previous combination of items reached the highest possible Alpha.

The Kappa coefficient showed minimal agreement in question 1 (p-value: 0.149), question 5 (p-value: 0.018), question 9 (p-value: 0.018), question B3 (p-value: 0.140), question B7 (p-value: 0.086) and question B9 (p-value: 0.140). In the other questions, the agreement was classified as perfect, as it reached p-value = 1 or there was no calculation possible because all the scores were identical, indicating total agreement between the judges.

### Hemodialysis

The 30 HD patients answered ten questions on the CKD questionnaire, and the measured Cronbach’s Alpha was 0.746; a result classified as adequate (> 0.7). That is, the questionnaire has minimum appropriate validation to be applied in other studies, as shown in Table 2.

**Table 2:** Cronbach’s alpha for epoch-rrt “interview protocol for patients with chronic kidney diseases on hemodialysis”

Statistics per item - Questionnaire por patients under hemodialysis (specific kidney questions)				
**Item**	**Mean value if item is deleted**	**Variance if item is deleted**	**Correlation corrected if item is deleted**	**Cronbach’s alpha if item is deleted**
Q 1_Health_General	41.63	186.24	0.084	0.754
Q 5_Frequency_dialysis	41.93	189.51	-0.111	0.756
Q 5_Hours_dialysis	40.93	187.79	0.233	0.753
Q 9_Stomach_full	39.13	146.95	0.392	0.732
Q 9_Tired_always	39.07	128.48	0.740	0.663
Q 9_Problems_sleep	39.07	136.27	0.567	0.697
Q 9_Itching_dry_skin	39.47	144.19	0.457	0.719
Q 9_Shortness_of_breath	41.27	137.10	0.673	0.680
Q 9_Lacking_apetite	40.80	154.37	0.454	0.718
Q 9_Pain_stomach	40.80	163.13	0.289	0.743

The calculated Kappa coefficient showed minimal agreement in question 1 (p-value: 0.189), question 9 (p-value: 0.086), question B3 (p-value: 0.018), question B7 (p-value: 0.086 )and question B9 (p-value: 0.140). In the other questions, the agreement was classified as perfect, as it reached a p-value equal to 1, or there was no calculation possible because all the scores were identical, indicating total agreement among the judges.

### Conclusions About Cronbach’s Alpha, Kappa Coefficient and CVI

The EPOCH-RRT questionnaires for PD and HD patients have an acceptable estimate to be replicated in other studies.

In patients without dialysis, the Cronbach’s Alpha was lower than 0.6, but, as previously mentioned, it would be inappropriate to use it due to its qualitative nature. It is therefore recommended that the EPOCH-RRT for non-dialysis patients be tested in other studies to see if adequate validation is possible or, alternatively, to include more discrete quantitative multiple-choice questions for patients to objectively answer on the impact of dialysis, chronic kidney disease and treatment. In the way it is presented, it does not seem to us that this questionnaire should be applied in studies that demand reliability. It is feasible to carry out a descriptive analysis of a group of patients undergoing conservative treatment, but it is not recommended to apply statistical tests or present it as a statistically validated instrument, because it is a questionnaire aimed at a qualitative analysis. The summary of Cronbach’s Alpha findings for the conservative treatment, HD and PD groups is shown in Table 3.

**Table 3:** Cronbach's alpha for epoch-rrt of the three groups of patients

Cronbach’s Alpha			
**Questionnaire**	**Conservative treatment**	**Peritoneal dialysis**	**Hemodialysis**
**Coefficient**	0.023	0.791	0.746
**Items**	2	8	10

For the 10 judges who evaluated the questionnaires, the Kappa coefficient showed perfect agreement in the vast majority of specific and general questions. Questions 1, B3, B7 and B9 generated minimal agreement in all questionnaires. Questions 5, 7 and 9 showed minimal agreement in some questionnaires. Disagreement occurred in the PD group questionnaire in question 5; in the conservative treatment group, in question 7; and in the DP and HD groups, in question 9.

The CVI obtained for the 3 questionnaires was 0.98, with an index > 0.8 being considered valid and an ideal > 0.9.

## Discussion

CKD has gained importance and been a matter of concern in Public Health in Brazil and around the world due to the increasing incidence and prevalence, the negative impact on health-related quality of life, that is high morbidity and mortality, in addition to personal, physical, emotional, spiritual, social and financial issues^38^.

In the last decades, technological advances have been observed in dialysis procedures, but the reality is that clinicians who work in primary care and other specialists who care for patients with impaired renal function are not always prepared to assist chronic kidney patients and refer them appropriately, enabling the necessary preparation for the stage where dialysis will be necessary^39^.

As highlighted earlier, there is a lack of national data on the participation of chronic kidney patients in their treatment choices. Generally, they start dialysis on an unscheduled basis, arriving at urgent or emergency services without knowledge of their disease and without any preparation for this new life-threatening situation^19^.

The lack of data and professional training cannot be a justification for inaction, and the current state must be recognized and overcome with strategies that allow an improvement in this dismal scenario.

There is no way to explain the importance of this study without placing the bioethical principle of autonomy, which is increasingly discussed in healthcare literature. This principle recommends that healthcare professionals or researchers, during their care or research practices, encourage and respect free will, values and preferences of each individual. Thus, the well-informed patient, interacting with the doctor and other healthcare professionals can decide about himself and his life condition^40^.

The principlism bioethics became better known in Brazil from the first regulations in 1996, which approved guidelines and regulatory norms for research involving human beings in the country, and in its preamble, it deals with the four principles presented as four basic references of bioethics.

An autonomous choice of patients involves educational and awareness processes, so much so that Guidelines/Guidelines and Opinions recommend that the choice between all treatment options for CKD should be planned and respect the conscious preference of patients, weighing risks and benefits^10^.

In our study, we achieved the main objective of translating and transculturally adapting the three questionnaires to the Brazilian context, and the instruments for PD and HD patients were also validated and reached reliable rates for their use.

A good questionnaire, whether qualitative or quantitative, should generate valid responses, be clear and easy to understand, well accepted by respondents and encourage participation and the provision of the desired information. As cultural differences may be present, these measures can be developed in two ways: developing a new measure or translating and culturally adapting a previously validated measure in another language^19^.

Thus, the option for choosing an instrument^14^ (questionnaires from the EPOCH-RRT study) that could be widely used in the national territory, once translated and validated, and already applied in another country, was precisely because of this scientific gap. Using them will be a unique opportunity to compare the results found with the original research developed in the United States of America.

Again, we must mention the fact that, for the most part, the questionnaires are essentially qualitative, with transcribed answers, in which qualitative analyzes are more coherent approaches than quantitative ones, as they assume a universe of senses, meanings, beliefs, values and attitudes, which dispense with numerical indicators^41^.

### Study Limitations

The main limitation of this study involving the translation and cross-cultural adaptation was the fact that the instruments were not exclusively quantitative, which, on the other hand, can be considered a strong point, since the two dialysis questionnaires were considered adequate, despite the more qualitative approach. The conservative treatment questionnaire may need more adaptations.

In the application of the instrument in the pilot project, the main limiting factor was the number of patients in the subgroups, which did not reach a total of at least 30 on PD and on conservative treatment. Another point is that HD patients undergo HD routine three times a week, which facilitates meetings and interviews. On the other hand, patients on PD had a longer time interval between consultations, which ended up extending in the covid-19 pandemic, so as not to expose them to greater risk. The same can be said of patients undergoing conservative treatment, whose elective consultations were suspended for a period and spaced at longer intervals.

### Clinical Implications

An innovative point of the study was to be based on questionnaires prepared with the collaboration of patients, family members and healthcare professionals in English and that could be reproduced in Brazil, after this study of translation and cross-cultural adaptation. Its application, even by fully viable means, such as digital media or telephone - means not tested by us -, which can result in increased knowledge and acquisition of new perspectives for all involved.

As far as we are aware, there are no similar questionnaires in Brazilian Portuguese that have the same purpose, whether original and developed here, or translated from another language.

## Conclusions

It was possible to translate, cross-culturally adapt and validate a tool suitable for hemodialysis and peritoneal dialysis patients that will help to identify and respect the patient’s priorities in their autonomous choice of the best treatment for themselves, and the gaps in shared decision-making.

## Supplementary Material

The following online material is available for this article:

Supplementary Material - Questionnaires used in the interview protocol of patients with chronic kidney disease.
