# Supplementary material for: Translation, cross-cultural adaptation and validation of the EPOCH-RRT questionnaire “Empowering Patients On Choices For Renal Replacement Therapy” for the Brazilian context
Source: J Bras Nefrol. 2022 Jun 27;45(1):67–76. doi: 10.1590/2175-8239-JBN-2021-0224en (PMC10139720; doi:10.1590/2175-8239-JBN-2021-0224en)
Supplement: Supplementary Material - [file 2175-8239-jbn-2021-0224-suppl.pdf]

Brazilian Journal of Nephrology
https://doi.org/10.1590/2175-8239-JBN-2021-0224en

# Supplementary material to article "Translation, transcultural adaptation, and validation of the EPOCH-RRT 'Empowering Patients on Choices for Renal Replacement Therapy' questionnaire for the Brazilian context"

## Questionnaires used in the interview protocol of patients with chronic kidney disease

### Item S2. Interview protocol for patients with chronic kidney disease on hemodialysis

**ID#____________**

**EPOCH-RRT (Empowering Patients on Choices for Renal Replacement Therapy)** [1]

**Interview protocol for patients with chronic kidney disease on hemodialysis**

Hi, [patient's name]. I'm [interviewer's name] and would like to ask you a few questions, since you've agreed to be interviewed for a study about people living with chronic kidney disease, called EPOCH. Is this is a good moment to talk? Great. The information collected during this interview is confidential and will be used to help other people with kidney disease. Know that your participation really matters to us.

[Interviewer: Read the consent term now, if applicable.]

1. I would like to start by asking a few questions about your overall health. You would describe your health, in most days, as… I will read some options to you: [circle the number that reflects the patient's answer]

Excellent……………………………………………1
Very good ……………………………………….. 2
Good………………………………………………...3
Fair……………………………………………... 4
Bad……………………………………………….5

[Probe:] Could you elaborate a little more about this? [Note to the Interviewer: try to understand how the patient arrived at such a health status. For example, is the interviewee's current health status connected to their kidney condition, other health conditions, or other events?]

2. I know that you have been told that you have a kidney condition. However, has a physician ever said to you that you also have diabetes, high blood pressure, or heart disease?

[Probe:] Do you have other health conditions?

3. Now, I would like to hear about your experience living with kidney disease.

[Probe:] When did you find out that you had a kidney condition?

[Probe:] How has this affected your everyday life?

[Probe:] Have you suffered with limitations in activities of daily living or been prevented from doing things that you like because of kidney disease?

[Probe:] Tell me a few examples of the things that you can no longer do because of your kidney condition.

4. Now, I would like you to think about your kidney condition in general. What bothers you the most as an outcome of having kidney disease?

[Probe:] What else bothers you?

How do you deal with your kidney condition?

A. I know that it takes a lot of effort to manage and take care of your kidney condition. What bothers you the most about it? [Interviewer: If the interviewee is not sure about what to say, give them examples such as fluid intake restrictions or having to undergo dialysis.]

B. How do you deal with it?

[Probe:] Does anything else bother you?

5. I learned that you are currently on hemodialysis, is that right?

A. Do you go to a dialysis center for treatment? How many times a week? How long do the sessions take?

B. How long have you been on hemodialysis for? [Note to the Interviewer: If the patient cannot tell you exactly how long they have been on hemodialysis for, ask them when they started undergoing hemodialysis.]

C. Have you ever been on peritoneal dialysis? [Note to the Interviewer: If yes, ask:] Could you tell me why you switched to hemodialysis?

D. Have you had a kidney transplant? [Note to the Interviewer: If not, probe:] Are you currently on a transplant waiting list? [If yes, probe:] Could you tell me a little more about the idea of receiving a kidney transplant? Do you know when it might happen?

6. Now, I would like to talk to you about the decision to undergo hemodialysis and how the treatment choice was made. Do you think that the decision to start hemodialysis was made primarily by you?

[Note to the Interviewer: If NOT, go to question A. If YES, go to question B.]

A. If the decision to start hemodialysis was not primarily yours, who made that decision for you?
[Probe:] Was it your kidney doctor, your partner, your children?
[Probe:] Can you tell me a little more about how this decision was made?
[Note to the Interviewer: Now go to question 7.]

B. When you made the decision, did anyone talk to you about different treatment options?

C. Do you think that you have received all necessary information about different treatment options including peritoneal dialysis, transplantation, or choosing not to be treated?

D. What led you to choose hemodialysis instead of peritoneal dialysis?

E. Should other people have been involved in the making of this decision? If so, who?

G. Which elements influenced your decision?
[Probe:] When you were trying to make this decision, did you talk to other patients about their experience? Was it useful?
[Probe:] Did you attend education classes or support group meetings? [Interviewer: If yes:] Did you participate with other people?

[Probe:] Which was more useful to you?

7. Now, I would like to explore matters that weighed in on your choice of treatment.

You must have had to consider a great deal of things while you were analyzing dialysis options. Could you tell me what was the most important element to you?

[Probe:] For example, was it important to have a schedule prepared at the dialysis center?

[Probe:] Did you think that it would be safer to go to a dialysis center to have dialysis administered to you?

[Probe:] What about keeping the highest level of independence possible?

[Probe:] Is spending time with other patients at a dialysis center important to you?

[Probe:] Were you worried about your appearance (for example, your physical appearance) after the start of dialysis?

[Probe:] Were you worried about how your dialysis treatment might affect other people?

[Probe:] Were matters related to quantity and quality of life important to you?

[Probe:] Is there anything else you might like to add?

8. Is there anything that you would like to have known before the start of hemodialysis that you are now aware of?

[Probe:] If you had been aware of these things, do you think that you might have chosen a different treatment option?

9. As you know, dialysis may produce side effects. I will read a list of things that other people have experienced, and I would like you to tell me if any of these things bothered you within the last four weeks. On a scale from 1 to 10, in which 1 means it did not bother you at all and 10 means you were extremely bothered, how much did the following things bother you? [Interviewer: Write the score given by the patient next to each of the phrases below.]

[Scale 1-10]

A. Sensation of having a full stomach /fullness ___________

B. Feeling tired all the time ___________

C. Trouble sleeping ___________

D. Pruritus, dry skin ___________

E. Breathlessness ___________

F. Lack of appetite ___________

G. Stomach ache or discomfort ___________

H. Would you like to add anything else? ___________

10. What advice would you give to someone trying to choose between different dialysis treatments? Which would you recommend and why?

11. Have we missed anything that you think should have been discussed?

**[General questions]**

Great! We are almost done. I just have to ask a few more basic questions. Some of them might sound personal, but they are important as we are trying to understand the complex issues faced by individuals with kidney disease. Your answers will be treated as confidential and will only be used for the purposes of this study. We can skip questions that you are not comfortable answering. Just let me know whenever this is the case. Let's begin.

B1: How old are you?

B2: How do you identify yourself in terms of race? [Interviewer: Do not read the categories below to the interviewee. Let them identify themselves in terms of race/ethnicity. The categories are not mutually exclusive; circle every applicable category.]

White...................................................... 1
Black…....................................................... 2
Brown…..................................................... 3
Yellow................................................... 4
Indigenous…............................................... 5
Other (please specify) ................... 6
Refused to answer or does not know....... 0

B3: What is your level of education? [Interviewer: Pick one]
Illiterate to elementary school graduate (0-9 year of schooling) ........... 1
Middle school or vocational school graduate .................. 2
Has not completed higher education........................................................ 3
Holder of an undergraduate degree.............................. 4
Holder of a graduate degree (Master's/Doctor's Degree)........................ 5

B4: What is your marital status? [Interviewer: Pick one and circle it.]

Single, never married.......................... 1

Married or in a state-registered domestic partnership ..................... 2

Divorced....…....................................... 3

Widow/widower......….…........................................ 4

Separated.......…....................................... 5

B5: In terms of living arrangements, please choose the one that best describes your current situation:

You live alone....…………………….....1

You live with other people…............. 2

[Interviewer: If the interviewee lives with other people, ask:] Who do you live with?

B6: Is anyone involved in providing care to you? [Interviewer: If yes, who?]

B7: What is your current employment situation? [Interviewer: Read the options below, stop as soon as the interviewee makes a choice, and circle the answer.]

Full-time job…………………... 1

Part-time job………………..…. 2

Retired...…………………………….……... 3

Housewife.......………………………….…. 4

Unemployed, looking for a job..........… 5

Unemployed, not looking for a job....... 6

On leave (sick pay) ................................7

B8: Now, let me ask you about your household income last year. Please include your income and the income earned by your partner and other members of your family that live with you at your home. I will read a number of income ranges. Please pick the one that better reflects your status. (Source: IBGE)

Up to 2 minimum wages/month….…………….......................... 1

More than 2 to 3.0 minimum wages/month……........…............…..2

More than 3.0 to 5.0 minimum wages/month………................…..3

More than 5.0 to 6.0 minimum wages/month…....………….....… 4

More than 6.0 to 8.0 minimum wages/month…...….......….………5

More than 8.0 to 10.0 minimum wages/month……..………..….. 6

More than 10.0 to 15.0 minimum wages/month…..…………….... 7

More than 15.0 to 20.0 minimum wages/month…..…………….... 8

More than 20.0 to 30.0 minimum wages/month…..…………….... 9

More than 30.0 minimum wages/month……………….........……10

Does not know…...................................................................….. 0

B9. Do you have health insurance?

Yes……...............1

No…………….....2 [If not, skip to B10]

Does not know…......... 3 [If does not know, skip to B10]

If yes, does your health insurance cover the costs of dialysis treatment?

Yes……...….….… 1 No……..… 2 Does not know……….. 0

Does your health insurance offer coverage for other costs, such as medication for kidney disease?

Yes……...….….… 1 No……..… 2 Does not know……….. 0

B10: Do you participate in educational programs for patients with kidney disease? If yes, which ones? Where and when?

B11: Do you participate in patient support groups (for example, talks with informed patients, old patients, or renal patient associations)?

That's it. These were the questions I had for you. Thank you for finding the time to talk to me. And thank you for contributing to this study.

**Item S1. Interview protocol for patients with chronic kidney disease off dialysis**

**ID#____________**

**EPOCH-RRT (Empowering Patients on Choices for Renal Replacement Therapy)** [1]

**Interview protocol for patients with chronic kidney disease OFF DIALYSIS**

Hi, [patient's name]. I'm [interviewer's name] and would like to ask you a few questions, since you've agreed to be interviewed for a study about people living with chronic kidney disease, called EPOCH. Is this is a good moment to talk? Great. The information collected during this interview is confidential and will be used to help other people with kidney disease. Know that your participation really matters to us.

[Interviewer: Read the consent term now, if applicable.]

1. I would like to start by asking a few questions about your overall health. You would describe your health, in most days, as… I will read some options to you: [circle the number that reflects the patient's answer]

Excellent……………………………………………1
Very good ………………………………………… 2
Good………………………………………………...3
Fair………………………………………... 4
Bad……………………………………………………5

Probe: Could you elaborate a little more about this? [Note to the Interviewer: try to understand how the patient arrived at such a health status. For example, is the interviewee's current health status connected to their kidney condition, other health conditions, or other events?]

2. I know that you have been told that you have a kidney condition. However, has a physician ever said to you that you also have diabetes, high blood pressure, or heart disease?
[Probe:] Do you have other health conditions?

3. Now, I would like to hear about your experience living with kidney disease.

[Probe:] When did you find out that you had a kidney condition?

[Probe:] How has this affected your everyday life?

[Probe:] Have you suffered with limitations in activities of daily living or been prevented from doing things that you like because of kidney disease?
[Probe:] Tell me a few examples of the things that you can no longer do because of your kidney condition.

4. Now, I would like you to think about your kidney condition in general. What bothers you the most as an outcome of having kidney disease?

   [Probe:] What else bothers you?

   How do you deal with your kidney condition?

   A. I know that it takes a lot of effort to manage and take care of your kidney condition. What bothers you the most about it? [Interviewer: If the interviewee is not sure about what to say, give them examples such as fluid intake restrictions or having to undergo dialysis.]

   B. How do you deal with it?

   [Probe:] Does anything else bother you?

5. Now, let me ask you a few questions about your future treatment options.

   A. Are you currently on a transplant waiting list?
   [Interviewer: Skip to question B below if the patient indicates that they will receive a transplant before starting dialysis.]

   B. As you probably know, the two most common types of dialysis are hemodialysis and peritoneal dialysis. Have you thought about which type of dialysis you might choose? I will read you a list with options. Please choose the one that best describes the stage you are in regarding choice of treatment. You would say that:
   [Circle the number for the answer]
   I have not thought about it yet……………………………………………..1
   I am considering my options ……………………………………..2
   I am close to making a decision………………………………………………..3
   I have made a decision, but am willing to reconsider………………………..4
   I have made a decision and will not reconsider…………………………5
   [Interviewer: If the answer was number 5, ask the patient which treatment they chose].

6. I imagine it must be very hard to choose the type of dialysis that is best for you. WHO do you think should be involved in making this decision?

   [Probe:] Do you think you and your physician should make this decision together?

   [Probe:] Would you rather have your physician make this decision alone?

   [Probe:] Should anyone else be involved?

7. I know that you have to consider many things when choosing between dialysis modes. For you, what is the most important element in this decision?

   [Probe:] For example, is it important for you to undergo dialysis at home?

   [Probe:] What about keeping the highest level of independence possible?

   [Probe:] How important is it for you to be able to work or go to school?

   [Probe:] Is it important to have flexibility in planning your days?

   [Probe:] Is it important to have a schedule prepared at a dialysis center?

   [Probe:] Are you worried about your appearance (for example, your physical appearance) after starting dialysis? Would having an arteriovenous (AV) fistula influence your choice of mode of dialysis?

   [Probe:] Do you think it would be safer to go to a dialysis center to have dialysis administered to you?

   [Probe:] Is spending time with other patients at a dialysis center important to you?

   [Probe:] Do you worry about how your dialysis treatment might affect other people?

   [Probe:] Are matters related to quantity and quality of life important to you?

   [Probe:] Is there anything else you might like to add?

8. [Interviewer: Some interviewees may not be prepared to discuss different treatment options or might not have thought about it yet. If this is the case, skip this question and move to number 9.] Which of these factors might be more important when considering hemodialysis? Which of these factors might make you consider peritoneal dialysis?

9. If you could talk to someone who has been through the process of deciding between dialysis modes, what would you ask them?

10. Is there anything that we have not discussed that you think we should talk about now?

**[General questions]**

Great! We are almost done. I just have to ask a few more basic questions. Some of them might sound personal, but they are important as we are trying to understand the complex issues faced by individuals with kidney disease. Your answers will be treated as confidential and will only be used for the purposes of this study. We can skip questions that you are not comfortable answering. Just let me know whenever this is the case. Let's begin.

B1: How old are you?

B2: How do you identify yourself in terms of race? [Interviewer: Do not read the categories below to the interviewee. Let them identify themselves in terms of race/ethnicity. The categories are not mutually exclusive; circle every applicable category.]

White....................................................... 1
Black…..................................................... 2
Brown…...................................................  3
Yellow..................................................  4
Indigenous….............................................. 5
Other (please specify) .................. 6
Refused to answer or does not know....... 0

B3: What is your level of education? [Interviewer: Pick one]
Illiterate to elementary school graduate (0-9 year of schooling) ........... 1
Middle school or vocational school graduate  .................  2
Has not completed higher education...................................................... 3
Holder of an undergraduate degree.............................. 4
Holder of a graduate degree (Master's/Doctor's Degree)....................... 5

B4: What is your marital status? [Interviewer: Pick one and circle it.]
Single, never married........................... 1
Married or in a state-registered domestic partnership .................... 2
Divorced.....…........................................ 3
Widow/widower......….…............................................ 4
Separated.......…...................................... 5

B5: In terms of living arrangements, please choose the one that best describes your current situation:

You live alone.....……………….………..1

You live with other people….......….. 2

[Interviewer: If the interviewee lives with other people, ask:] Who do you live with?

B6: Is anyone involved in providing care to you? [Interviewer: If yes, who?]

B7: What is your current employment situation? [Interviewer: Read the options below, stop as soon as the interviewee makes a choice, and circle the answer.]

Full-time job………….……... 1

Part-time job………….…..…. 2

Retired...…………………………….……... 3

Housewife.......…………………….……….… 4

Unemployed, looking for a job..........… 5

Unemployed, not looking for a job….... 6

On leave (sick pay) ................................7

B8: Now, let me ask you about your household income last year. Please include your income and the income earned by your partner and other members of your family that live with you at your home. I will read a number of income ranges. Please pick the one that better reflects your status. (Source: IBGE)

Up to 2 minimum wages/month….……………….......................... 1

More than 2 to 3.0 minimum wages/month……........…..........….…2

More than 3.0 to 5.0 minimum wages/month……………..........….…3

More than 5.0 to 6.0 minimum wages/month…....………………......… 4

More than 6.0 to 8.0 minimum wages/month…....…......…..………5

More than 8.0 to 10.0 minimum wages/month..……..……….…... 6

More than 10.0 to 15.0 minimum wages/month…..………….…... 7

More than 15.0 to 20.0 minimum wages/month…..………….…... 8

More than 20.0 to 30.0 minimum wages/month…..………….…... 9

More than 30.0 minimum wages/month………………..........….…10

Does not know…...............................................................….. 0

B9. Do you have health insurance?

Yes……...............1

No…………..…. 2 [If not, skip to B10]

Does not know…..…… 3 [If does not know, skip to B10]

If yes, does your health insurance cover the costs of dialysis treatment?

Yes…….....….…. 1 No……..… 2 Does not know………. 0

Does your health insurance offer coverage for other costs, such as medication for kidney disease?

Yes…….....….…. 1 No……..… 2 Does not know………. 0

B10: Do you participate in educational programs for patients with kidney disease? If yes, which ones? Where and when?

B11: Do you participate in patient support groups (for example, talks with informed patients, old patients, or renal patient associations)?

That's it. These were the questions I had for you. Thank you for finding the time to talk to me. And thank you for contributing to this study.

**Item S2. Interview protocol for patients with chronic kidney disease on peritoneal dialysis**

ID#____________

**EPOCH-RRT (Empowering Patients on Choices for Renal Replacement Therapy)** [1]

**Interview protocol for patients with chronic kidney disease on peritoneal dialysis**

Hi, [patient's name]. I'm [interviewer's name] and would like to ask you a few questions, since you've agreed to be interviewed for a study about people living with chronic kidney disease, called EPOCH. Is this is a good moment to talk? Great. The information collected during this interview is confidential and will be used to help other people with kidney disease. Know that your participation really matters to us..

[Interviewer: Read the consent term now, if applicable.]

1. I would like to start by asking a few questions about your overall health. You would describe your health, in most days, as… I will read some options to you: [circle the number that reflects the patient's answer]

Excellent……………………………………………1
Very good ………………………………………… 2
Good………………………………………………....3
Fair…………………………………………... 4
Bad………………………………………………5

[Probe:] Could you elaborate a little more about this? [Note to the Interviewer: try to understand how the patient arrived at such a health status. For example, is the interviewee's current health status connected to their kidney condition, other health conditions, or other events?]

2. I know that you have been told that you have a kidney condition. However, has a physician ever said to you that you also have diabetes, high blood pressure, or heart disease?

[Probe:] Do you have other health conditions?

3. Now, I would like to hear about your experience living with kidney disease.

[Probe:] When did you find out that you had a kidney condition?
[Probe:] How has this affected your everyday life?
[Probe:] Have you suffered with limitations in activities of daily living or been prevented from doing things that you like because of kidney disease?
[Probe:] Tell me a few examples of the things that you can no longer do because of your kidney condition.

4. Now, I would like you to think about your kidney condition in general. What bothers you the most as an outcome of having kidney disease?

[Probe:] What else bothers you?

How do you deal with your kidney condition?

C. I know that it takes a lot of effort to manage and take care of your kidney condition. What bothers you the most about it? [Interviewer: If the interviewee is not sure about what to say, give them examples such as fluid intake restrictions or having to undergo dialysis.]

D. How do you deal with it?

[Probe:] Does anything else bother you?

5. I learned that you are currently on peritoneal dialysis, is that right?

A. What dialysis mode are you on and how do you perform changes?

How long have you been on peritoneal dialysis?

B. Have you ever been on hemodialysis? [Note to the Interviewer: If yes, ask:] Could you tell me why you decided to switch to peritoneal dialysis?

C. Have you had a kidney transplant? [Note to the Interviewer: If not, probe:] Are you currently on a transplant waiting list? [If yes, probe:] Could you tell me a little more about the idea of receiving a kidney transplant? Do you know when it might happen?

6. Now, I would like to talk to you about the decision to undergo peritoneal dialysis and how the treatment choice was made. Do you think that the decision to start peritoneal dialysis was made primarily by you?

[Note to the Interviewer: If NOT, go to question A. If YES, go to question B.]

A. If the decision to start peritoneal dialysis was not primarily yours, who made that decision for you?

[Probe:] Was it your kidney doctor, your partner, your children?

[Probe:] Can you tell me a little more about how this decision was made?

[Note to the Interviewer: Now go to question 7.]

B. When you made the decision, did anyone talk to you about different treatment options?

C. Do you think that you have received all necessary information about different treatment options including peritoneal dialysis, transplantation, or choosing not to be treated?

D. What led you to choose peritoneal dialysis instead of hemodialysis?

E. In addition to you, who else was involved in the making of this decision?

F. Should other people have been involved in the making of this decision? If so, who?

G. Which elements influenced your decision?

[Probe:] When you were trying to make this decision, did you talk to other patients about their experience? Was it useful?

[Probe:] Did you attend education classes or support group meetings? [Interviewer: If yes:] Did you participate with other people?

[Probe:] Which was more useful to you?

7. Now, I would like to explore matters that weighed in on your choice of treatment.

You must have had to consider a great deal of things while you were analyzing dialysis options. Could you tell me what was the most important element to you?

[Probe:] For example, how important was it to undergo dialysis in the privacy and comfort of your home instead of having to go to a clinic three times a week?

[Probe:] What about keeping the highest level of independence possible?

[Probe:] How important is it for you to be able to work or go to school?

[Probe :] Is it important to have flexibility in planning your days?

[Probe:] Were you worried about your appearance (for example, your physical appearance) after starting dialysis?

[Probe:] Did you worry about how your dialysis treatment might affect other people?

[Probe:] Are matters related to quantity and quality of life important to you?

[Probe:] Is there anything else you might like to add?

8. Is there anything that you would like to have known before the start of peritoneal dialysis that you are now aware of?

[Probe:] If you had been aware of these things, do you think that you might have chosen a different treatment option?

9. As you know, dialysis may produce side effects. I will read a list of things that other people have experienced, and I would like you to tell me if any of these things bothered you within the last

four weeks. On a scale from 1 to 10, in which 1 means it did not bother you at all and 10 means you were extremely bothered, how much did the following things bother you? [Interviewer: Write the score given by the patient next to each of the phrases below.] [Scale 1-10]

A. Sensation of having a full stomach /fullness ___________
B. Feeling tired all the time ___________
C. Trouble sleeping ___________
D. Pruritus, dry skin ___________
E. Breathlessness ___________
F. Lack of appetite ___________
G. Stomach ache or discomfort ___________
H. Would you like to add anything else? ___________

10. What advice would you give to someone trying to choose between different dialysis treatments? Which would you recommend and why?

11. Have we missed anything that you think should have been discussed?

**[General questions]**

Great! We are almost done. I just have to ask a few more basic questions. Some of them might sound personal, but they are important as we are trying to understand the complex issues faced by individuals with kidney disease. Your answers will be treated as confidential and will only be used for the purposes of this study. We can skip questions that you are not comfortable answering. Just let me know whenever this is the case. Let's begin.

B1: How old are you?

B2: How do you identify yourself in terms of race? [Interviewer: Do not read the categories below to the interviewee. Let them identify themselves in terms of race/ethnicity. The categories are not mutually exclusive; circle every applicable category.]

White....................................................... 1
Black…..................................................... 2
Brown….................................................... 3
Yellow.................................................... 4
Indigenous…............................................. 5

Other (please specify) ……………… 6

Refused to answer or does not know….… 0

B3: What is your level of education? [Interviewer: Pick one]

Illiterate to elementary school graduate (0-9 year of schooling) ……….. 1

Middle school or vocational school graduate …………….. 2

Has not completed higher education…………………………………………… 3

Holder of an undergraduate degree………………………… 4

Holder of a graduate degree (Master's/Doctor's Degree)…………………… 5

B4: What is your marital status? [Interviewer: Pick one and circle it.

Single, never married……………………… 1

Married or in a state-registered domestic partnership ……………….. 2

Divorced……………………………………… 3

Widow/widower…………………………………………… 4

Separated……………………………………… 5

B5: In terms of living arrangements, please choose the one that best describes your current situation:

You live alone……………………………1

You live with other people…………… 2

[Interviewer: If the interviewee lives with other people, ask:] Who do you live with?

B6: Is anyone involved in providing care to you? [Interviewer: If yes, who?]

B7: What is your current employment situation? [Interviewer: Read the options below, stop as soon as the interviewee makes a choice, and circle the answer.]

Full-time job…………………... 1

Part-time job………………….… 2

Retired…………………………………….….... 3

Housewife……………………………………… 4

Unemployed, looking for a job…………. 5

Unemployed, not looking for a job….… 6

On leave (sick pay) ……………………………7

B8: Now, let me ask you about your household income last year. Please include your income and the income earned by your partner and other members of your family that live with you at your home. I will read a number of income ranges. Please pick the one that better reflects your status. Source: IBGE

Up to 2 minimum wages/month……………………............................ 1
More than 2 to 3.0 minimum wages/month…….........…........….…2
More than 3.0 to 5.0 minimum wages/month…………..............…..3
More than 5.0 to 6.0 minimum wages/month…....………….......… 4
More than 6.0 to 8.0 minimum wages/month…...…......…..………5
More than 8.0 to 10.0 minimum wages/month..……...…………..….. 6
More than 10.0 to 15.0 minimum wages/month…..…………….…... 7
More than 15.0 to 20.0 minimum wages/month…..………….…... 8
More than 20.0 to 30.0 minimum wages/month…..…………….…... 9
More than 30.0 minimum wages/month……………….........…..10
Does not know…....................................................................…..0

B9. Do you have health insurance?

Yes…….............1

No……………... 2 [If not, skip to B10]

Does not know…....... 3 [If does not know, skip to B10]

If yes, does your health insurance cover the costs of dialysis treatment?

Yes…….....…….… 1

No………………. 2

Does not know….….… 0

Does your health insurance offer coverage for other costs, such as medication for kidney disease?

Yes…………..…… 1

No……………. 2

Does not know…......0

B10: Do you participate in educational programs for patients with kidney disease? If yes, which ones? Where and when?

B11: Do you participate in patient support groups (for example, talks with informed patients, old patients, or renal patient associations)?

That's it. These were the questions I had for you. Thank you for finding the time to talk to me. And thank you for contributing to this study.

## REFERENCES

1. Dahlerus C, Quinn M, Messersmith E, Lachance L, Subramanian L, Perry E, et al. Patient perspectives on the Choice of Dialysis Modality: Results from the empowering patients on choices for renal replacement therapy (EPOCH-RRT) Study. Am J Kidney Dis. 2016;68(6):901-910.
